# Targeting the Interplay between Epithelial-to-Mesenchymal-Transition and the Immune System for Effective Immunotherapy

**DOI:** 10.3390/cancers11050714

**Published:** 2019-05-24

**Authors:** Rama Soundararajan, Jared J. Fradette, Jessica M. Konen, Stacy Moulder, Xiang Zhang, Don L. Gibbons, Navin Varadarajan, Ignacio I. Wistuba, Debasish Tripathy, Chantale Bernatchez, Lauren A. Byers, Jeffrey T. Chang, Alejandro Contreras, Bora Lim, Edwin Roger Parra, Emily B. Roarty, Jing Wang, Fei Yang, Michelle Barton, Jeffrey M. Rosen, Sendurai A. Mani

**Affiliations:** 1 Department of Translational Molecular Pathology, The University of Texas MD Anderson Cancer Center, Houston, TX 77030, USA; 2 Department of Thoracic/Head-Neck Medical Oncology, The University of Texas MD Anderson Cancer Center, Houston, TX 77030, USA; 3 Department of Breast Medical Oncology, The University of Texas MD Anderson Cancer Center, Houston, TX 77030, USA; 4 Department of Molecular and Cellular Biology, Baylor College of Medicine, Houston, TX 77030, USA; 5 Department of Chemical and Biomolecular Engineering, University of Houston, Houston, TX 77204, USA; 6 Departments of Melanoma Medical Oncology–Research and Translational Molecular Pathology, The University of Texas MD Anderson Cancer Center, Houston, TX 77030, USA; 7 Department of Integrative Biology and Pharmacology, The University of Texas Health Sciences Center, Houston, TX 77030, USA; 8 Department of Pathology (Anatomical), The University of Texas MD Anderson Cancer Center, Houston, TX 77030, USA; 9 Department of Bioinformatics and Computational Biology, The University of Texas MD Anderson Cancer Center, Houston, TX 77030, USA; 10 Department of Epigenetics and Molecular Carcinogenesis, The University of Texas MD Anderson Cancer Center, Houston, TX 77030, USA

**Keywords:** CD8 T Cells, immune blockade, NSCLC, reversal of EMT, tumor microenvironment, tumor plasticity, TNBC

## Abstract

Over the last decade, both early diagnosis and targeted therapy have improved the survival rates of many cancer patients. Most recently, immunotherapy has revolutionized the treatment options for cancers such as melanoma. Unfortunately, a significant portion of cancers (including lung and breast cancers) do not respond to immunotherapy, and many of them develop resistance to chemotherapy. Molecular characterization of non-responsive cancers suggest that an embryonic program known as epithelial-mesenchymal transition (EMT), which is mostly latent in adults, can be activated under selective pressures, rendering these cancers resistant to chemo- and immunotherapies. EMT can also drive tumor metastases, which in turn also suppress the cancer-fighting activity of cytotoxic T cells that traffic into the tumor, causing immunotherapy to fail. In this review, we compare and contrast immunotherapy treatment options of non-small cell lung cancer (NSCLC) and triple negative breast cancer (TNBC). We discuss why, despite breakthrough progress in immunotherapy, attaining predictable outcomes in the clinic is mostly an unsolved problem for these tumors. Although these two cancer types appear different based upon their tissues of origin and molecular classification, gene expression indicate that they possess many similarities. Patient tumors exhibit activation of EMT, and resulting stem cell properties in both these cancer types associate with metastasis and resistance to existing cancer therapies. In addition, the EMT transition in both these cancers plays a crucial role in immunosuppression, which exacerbates treatment resistance. To improve cancer-related survival we need to understand and circumvent, the mechanisms through which these tumors become therapy resistant. In this review, we discuss new information and complementary perspectives to inform combination treatment strategies to expand and improve the anti-tumor responses of currently available clinical immune checkpoint inhibitors.

## 1. Rethinking Cancer Therapy Development

Over the last decade, pivotal technological and clinical advances have dramatically impacted the survival of some cancer patients. This began with rapid and efficient genomic sequencing that markedly expanded our knowledge beyond the scaffold delivered by the initial Human Genome Project into the realm of tumor-driving mutations, some of which are seen in only a small fraction of cancers. Primarily due to the advancements facilitated by these data, the majority of new oncologic agents approved today are biologically targeted as opposed to cytotoxics.

Among various cancers, non-small cell lung cancer (NSCLC) and triple-negative breast cancer (TNBC) are the first and fourth most common causes of cancer-related mortality in the U.S. [1]. These cancers possess many similarities based on molecular classification and gene expression analyses despite their distinct tissues of origin [2]. Epithelial cells are the heartiest of embryologically derived layers. Topologically, they are external-facing barrier cells that are therefore endowed with protective mechanisms including membrane transport channels, tight junctions, and built-in plasticity mechanisms for adaptive responses to numerous insults even in their benign states—this makes them formidable enemies when they undergo malignant transformation. Targeted therapies for oncogenic aberration in lung (e.g., EGFR and ALK kinase inhibitors) and breast (e.g., HER2 therapies) cancer have improved survival, but have not resulted in cures for all patients. In advanced NSCLC, responsible for the largest number of cancer-caused deaths in the U.S., it has now become standard clinical practice in metastatic disease to obtain genomic sequencing, including for EGFR or ALK gene mutations/rearrangements in order to select drugs that significantly improve survival. Assays for HER2 overexpression and/or gene amplification are standard for every breast cancer case.

As the cost of gene sequencing has dropped, methods of “deeper” sequencing with accuracy to single-cell resolution have been developed. Single-cell sequencing has revealed that tumors are composed of genomically and transcriptionally diverse cells. Clonal selection and adaptive responses lead to drug resistance, immune escape, and tumor dissemination. Single-cell sequencing using topographic spatial information in tissue sections of breast ductal carcinoma and associated metastases revealed the direct genomic lineage between in situ and invasive subpopulations, demonstrating that such diversity is an early phenomenon that allows for pre-invasive selection and likely explains the complex constellation of phenotypes that cancer cells possess from the outset [3,4,5]. Innovations in both genomics and proteomic analytic techniques, increasingly being applied pre- and post-treatment, have also revealed extensive rewiring of cellular networks associated with tumor progression, metastasis, and drug resistance. These adaptive changes can be mediated by epigenetic modifications or microRNAs (miRNAs), and other pre- and post-transcriptional, post-translational and tumor microenvironmental events [6,7,8]. Each of these processes represents therapeutic opportunities that can be tested in preclinical models and ultimately in the clinic.

Importantly, interactions of malignant cells with the tumor microenvironment, including immune cells, the vascular system, stromal cells, and stem cells of different lineages contribute to phenotypic plasticity driven by de-differentiation, increased stem-cell behavior, and cells that have undergone the epithelial-mesenchymal transition (EMT) [9]. These events can generate overlapping yet distinct functional compartments that escape natural immunity and subsequent treatment [10,11,12]. In fact, the characteristics of immunoevasion, energy reprogramming, and “collusion” with the tumor microenvironment are recognized as the next generation of the hallmarks of cancer [13,14]. These interacting traits are part of a multi-dimensional construct that necessitates creative approaches to therapeutic intervention. RECIST (Response Evaluation Criteria in Solid Tumors) is currently used for evaluating objective treatment response for the majority of clinical trials. Clearly, based upon the above discussion RECIST as the sole endpoint for clinical trials is clearly not sufficient as revealed by the extensive intratumoral and microenvironmental heterogeneity observed in both primary and metastatic disease. Instead, better criteria, including the inclusion of the expression and spatial localization of specific biomarkers, both tumor intrinsic and in the microenvironment, and the detailed evaluation of residual disease will be required to develop more efficacious cancer therapies.

## 2. Tumors Responsive to Immunotherapy

Though immune responses to cancer have been recognized for decades, the general consensus was that most were insufficient to eradicate established cancers due to immune suppression mediated through several mechanisms. Immune checkpoint blockade therapies (ICBT) are revolutionizing and rapidly emerging as a game-changing approach in the treatment of many cancer types. ICBTs are remarkably effective and approved for several cancer types including metastatic melanoma and non-small cell lung cancer [15,16,17]. The premise of cancer immune checkpoint therapy (ICBT) is that harnessing the patient’s natural cancer immune defense system leads these cells to selectively search out and destroy cancer cells [18]. By targeting negative regulators of T cell activity, they unleash anti-tumor immunity.

In September 2014, the anti-PD-1 antibody pembrolizumab was the first agent targeting the PD-1/PD-L1 interaction to receive FDA approval for metastatic melanoma. There are data suggesting that PD-L1 expression on the tumor may be a biomarker predicting response to this class of therapy [19,20]. PD-L1 expression was also seen in 20% of TNBC tumors suggesting that targeting PD-1 or PD-L1 may have therapeutic benefit in TNBC [21]. Single-agent trials of PD-1 or PD-L1 inhibition in TNBC have demonstrated response rates of 5–19%, with some patients experiencing prolonged, durable responses. [22,23,24]. Some of these studies required ≥1% expression of PD-L1 as an entry criterion, while others did not. Responses have been seen in tumors that lack PD-L1 expression, and thus far, the data do not definitively suggest that PD-L1 expression is required for single agent biologic activity of immune checkpoint inhibitors in TNBC.

Tumor cell killing by cytotoxic chemotherapy like anthracyclines and carboplatins can facilitate immunogenic cell death and facilitate an adaptive immune response [25]. Invigorating tumor-specific T-cell immunity in this setting by inhibiting PD-L1/PD-1 signaling may result in deeper and more durable responses compared to standard chemotherapy alone.

Supporting this hypothesis, a randomized phase III trial of nab-paclitaxel+/− atezolizumab for the first line treatment of metastatic TNBC was the first to show a benefit for immunotherapy [26]. Notably, unlike single agent checkpoint inhibitor trials, benefit was only seen in the group of tumors that expressed PD-L1 within the immune infiltrate and, as such, the combinatorial strategy has been recently FDA approved for unresectable locally advanced or metastatic TNBC patients who have PD-L1 stained tumor-infiltrating immune cells covering at least ≥1% of the tumor area (https://www.fda.gov/Drugs/InformationOnDrugs/ApprovedDrugs/ucm633065.htm).

In lung cancer, immunotherapy has rapidly become a standard part of therapy for patients with advanced cancers, with several anti-PD1 and/or anti-PDL1 drugs approved by the FDA for non-small cell lung cancer (NSCLC). Specifically, patients with metastatic NSCLC and PDL1 tumor proportion score of at least 50% can be treated with front-line single agent pembrolizumab, while patients with PDL1 levels below 50% can receive immunotherapy in combination with chemotherapy (i.e., carboplatin-pemetrexed-pembrolizumab, carboplatin-taxane-pembrlizumab combinations) in the frontline setting or one of three single-agent immunotherapy agents (pembrolizumab, nivolumab, or atezolizumab) in the second-line setting for relapsed disease [27,28,29]. However, despite the enthusiasm for immunotherapy for lung cancer, many patients do not receive clinical benefit from these agents, and even in those who do respond initially, therapeutic resistance can develop over time.

The renaissance of immunotherapy with the discovery of checkpoints and other modulators of immunity has brought on a new era in cancer therapeutics. In many types of non-epithelial malignancies, immune checkpoint blockade therapy (ICBT) achieves long-term remissions. However, ICBT is often ineffective in lung cancer and is rarely successful for breast cancer, for which this therapy remains investigational [30,31]. In addition to intrinsic resistance to ICBT, acquired resistance, defined as clinical progression after an initial response or prolonged stability, is also seen with targeted or cytotoxic therapy.

## 3. Role of EMT in Immune Evasion

**Figure 1:**
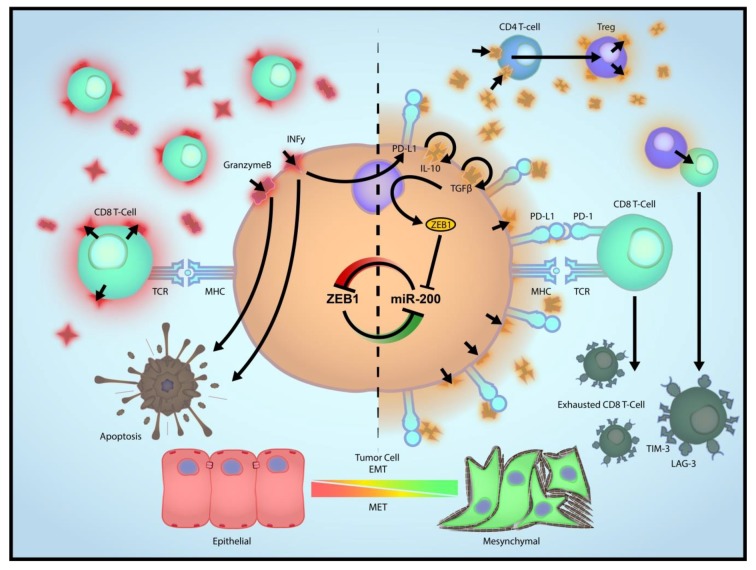
Tumor cell EMT drives multiple parallel pathways of immune suppression. Epithelial tumor cells are more sensitive to the effects of CD8+ effector cytotoxic T cells. Mesenchymal tumor cells, as illustrated by high expression of the transcriptional repressor ZEB1 and concordant suppression of the microRNA-200 family, express increased levels of PD-L1, immune suppressive cytokines (e.g., TGFβ), and enhanced recruitment of immune suppressive cells (e.g., CD4+ T regulatory cells). These EMT-directed changes produce exhaustion of CD8+ T cells or suppress their recruitment into the tumor microenvironment. CD8 T cell: CD8+ effector cytotoxic T cells; Treg: Regulatory T cell.

EMT directly regulates expression of PD-L1 and is associated with several other checkpoint ligands [32,33] (Figure 1). Thus, EMT is expected to induce checkpoint-dependent resistance to anti-tumor immunity. Due to the redundancy of the multiple checkpoints, EMT may render cancer cells non-responsive to therapies targeting one or few checkpoints (e.g., anti-PD-L1 and anti-CTLA4). Due to the limited scope of this review, we are unable to discuss many articles here; however, the reader is referred some notable publications in this context [12,34,35,36,37,38]. Additionally, EMT drives the recruitment of tumor-associated macrophages, which may, in turn, mediate resistance to immunotherapies [39,40]. This may be achieved through direct regulation of cytokinome of cancer cells (e.g., CCL2). The immunosuppression by macrophages, especially the alternatively activated macrophages (M2), has been extensively studied and involves several mechanisms [41]. The tumor suppressive and tumor promoting effects of EMT shift the balance between macrophages and neutrophils. Thus, inhibition of EMT, while overcoming immunosuppression by cancer cells and macrophages, may coincidentally cause accumulation of a type of neutrophils in some tumors defined as myeloid-derived suppressor cells leading to an “escape” pathway from anti-EMT treatment. Therefore, it is important to examine the clinical correlation between EMT and the entire immune microenvironment, including the myeloid cell compartment.

## 4. EMT as a Driver of Immune Escape from ICBT

Despite the presence of an immune response, some tumors continue to grow, a process that has been referred to as “immune escape”. Tumor cell escape can occur through multiple different mechanisms [42]. Tumor cells themselves promote an immunosuppressive microenvironment by producing suppressive cytokines including TGF-β, VEGF, or indoleamine 2,3-dioxygenase. The tumor microenvironment also contains immune cells such as regulatory T cells and myeloid-derived suppressor cells that function to suppress the immune response. At the individual tumor cell level, alterations leading to decreased immune recognition (such as loss of tumor antigens, downregulation of major histocompatibility complex molecules, or loss of antigen processing function within the tumor cell), or increased resistance to the cytotoxic effects of immunity (such as via induction of anti-apoptotic mechanisms) can promote tumor growth. Finally, tumor cells can upregulate T cell-inhibitory molecules such as PD-L1, which is why activating the immune system for therapeutic benefit in cancer is an area of active investigation. For a more elaborate discussion on this topic, we refer readers to some recent reviews [10,43,44].

We and others have previously described epithelial-mesenchymal transition (EMT) as a frequent mechanism of de novo and acquired therapeutic resistance in mammary tumor cells, as well as all subtypes of NSCLC, including lung cancers with oncogenic driver mutations in EGFR and KRAS [45,46,47,48]. Furthermore, we and others have demonstrated that EMT upregulates expression of PD-L1 in murine and human NSCLC and directly leads to CD8+ T cell exhaustion and immunosuppression. To facilitate the study of EMT across large cohorts, our group identified a robust, platform-independent lung cancer EMT gene expression signature valid in lung cancer cell lines and NSCLC patient tumors [45]. The signature was then further refined to develop a patient-based, pan-cancer EMT signature using 1934 patient tumors from multiple solid tumor types including breast, lung, colon, and other common cancers [2]. The Lung EMT and Pan-Cancer EMT signatures are highly correlated with mesenchymal cancers from distinct tumor types showing striking similarities in their molecular profiles. Using the EMT signatures, an individual cancer cell line or tumor can be scored for the degree to which it has undergone EMT. These mRNA-based EMT signature scores are easy to measure, and are highly correlated with other established EMT markers (such as expression of E-cadherin protein) as well as with other factors known to regulate EMT (e.g., miR200 family and the transcription factor ZEB1). The applicability of EMT scores to predict lung cancer has been mirrored in breast cancer patients as well [49]. Residual breast cancers after conventional chemotherapy were shown to exhibit mesenchymal features. In addition to applying the EMT scores in our lung cancer research, we generated EMT scores for multiple TCGA cohorts, which has allowed integrated analyses of the relationships between EMT and other molecular or immune data profiles (i.e., miRNA and methylation profiles, iCLUSTER data) [32,50,51].

In addition to its broad tumor cell-autonomous impact, EMT in breast cancer also profoundly alters the microenvironment landscape, especially immune cell constituents (Figure 1). Macrophages and neutrophils are key modulators of the tumor microenvironment [52,53]. Recent evidence suggests a strong correlation between EMT and a switch from a neutrophil- enriched immune profile to a macrophage-dominant profile. Tumors exhibiting an epithelial-like phenotype tend to have local and systemic accumulation of neutrophils. In contrast, tumors with mesenchymal features are predominantly infiltrated with macrophages that are often but not always polarized to the M2 (alternatively activated) status. These two categories of tumors are defined as a neutrophil-enriched subtype (NES) and a macrophage-enriched subtype (MES), respectively. Inducible expression of miR-200, a master regulator of EMT, shifts the macrophage/neutrophil balance, supporting the causal role of EMT in determining the myeloid cell profile of the tumor microenvironment.

Mechanistically, several transcriptional suppressors regulate EMT, including the two-handed zinc-finger δEF1 family transcription factors ZEB1 and ZEB2 [54,55,56,57]. ZEB1/2 binds to E-box regions in the promoters of key epithelial differentiation genes such as E-cadherin, and transcriptionally suppress their expression [58]. ZEB1/2 also regulates the miR-200 family of miRNAs miR-141, 200a/b/c, and 429 that are broadly expressed in normal epithelial cells [59]. miR-200 is a master EMT regulator, governed by a double-negative feedback loop with the ZEB repressors [60,61,62,63,64] and regulated by multiple EMT inducers (e.g., TGFβ) [61,62]. miR-200 loss has been linked to stem-like features and chemoresistance [65,66]. Evidence from several epithelial tumor types, including lung and breast, implicates miR-200 dysregulation in disease progression [64,67,68]. Using the KP mouse model and a panel of human NSCLC cell lines, we have demonstrated that the miR-200/ZEB1 feedback loop is a critical regulatory axis that determines metastatic potential [69,70] by controlling global mRNA changes in an invasive subset of tumor cells, modulating matrix-dependent tumor activation and invasion [70,71,72,73].

## 5. Relationship between CD8 T Cells and EMT, and Impact of EMT on ICBT

In breast cancer, there is a strong correlation between tumor-infiltrating T and B cells and favorable prognostic outcome or therapeutic responses (standard therapies) [18,74,75,76,77]. Many standard-of-care therapies require the immune system to exert their effects. The most prominent example is anti-HER2 treatment with trastuzumab, which heavily relies on functional host immunity [78]. Furthermore, it has recently been recognized that the response of some chemotherapy regimens is achieved by their impact on immunosuppressive cells [79,80,81]. Thus, it appears that the immune system in breast cancer patients retains the potential of fighting cancer. Ongoing clinical trials suggest that although some breast cancer patients may benefit from ICBT [22,82], the percentage is disappointingly low. Possible mechanisms of resistance include low level or ineffective neoantigens [83,84] and/or the enrichment of immunosuppressive cells [85,86], and the elaboration of immunosuppressive cytokines (e.g., IL-10, TGF-β), all of which may result in a scarcity of functional cytotoxic T cells. Therefore, to improve the efficacy of immunotherapy it will be critically important to tackle these potential mechanisms. In the following section, we will focus on the latter: the immunosuppressive microenvironment that prevents anti-tumor immunity.

ICBT enhances anti-tumor responses by increasing the activity of the cytotoxic CD8+ T lymphocyte subpopulations; these cells are key players in the effector functions of adaptive immunity [87]. In addition to secreting chemokines and cytokines, tumor-specific T cells interact through T cell receptors with the major histocompatibility complex (MHC) on antigen-presenting cells, which in turn triggers a signaling cascade resulting in the death of target cells [88,89]. Currently, adoptive T cell-transfer, oncolytic viruses, cancer vaccines, T cell co-stimulatory agonists and monoclonal antibodies are used either alone or in combination with ICBT [90]. Checkpoint regulators of immune activation help maintain immune homeostasis and prevent autoimmunity. However, the immune checkpoint pathways are frequently activated in cancer to suppress the nascent anti-tumor immune response. A combination of immune checkpoint inhibitors namely PD-1 and CTLA-4 can effectively kill cancer cells because they function primarily through complementary mechanisms [91]. When CD8+ T cells recognize self-antigen on tumor cells, they fail to kill cancer cells; this immunological tolerance is a drawback of the ICBT [92]. In addition, CD8+ T cell exhaustion due to chronic exposure to antigens negatively affects the efficacy of ICBT [93].

Tumor cells evade immunosurveillance by altering their phenotype via immunoediting, and it is known that immuno-edited tumors display properties of cells that have undergone EMT [94]. Activated CD8^+^ T cells, macrophages, and several other immune cell types produce TGF-β, a crucial promoter of EMT [95,96,97]. We previously demonstrated that two key EMT factors that are also markers of cancer stem cells (CSCs), FOXC2 and Twist, are necessary for the process of breast carcinoma metastasis [57,98]. Early evidence suggested an antitumor response of CD8^+^ T cells delayed metastasis and eliminated disseminated tumor cells (DTC) of P815 mastocytoma [99]. In a melanoma mouse model, CD8^+^ T cells are involved in maintaining DTC dormancy in visceral organs like the lungs and the reproductive tract, thereby preventing overt metastasis and limiting disease progression [32]. CD8^+^ T cells are also known to inhibit tumor growth thus prolonging the survival of experimental mice by selectively targeting cancer stem cells (CSCs) [100]. CD8^+^ T cell suppression within the tumor microenvironment is dependent on PD-L1 regulation on tumor cells via a pathway involving the microRNA miR-200 and the transcription factor ZEB1; these molecules are the links between EMT, CD8^+^ T cell exhaustion, and tumor suppression [32]. Furthermore, the inhibition of breast cancer onset and progression is inhibited by CD8^+^ T cells and natural killer (NK) cells due to increased cytotoxic activity mediated by the protein TIPE2 [101]. Although EMT leads to up-regulation of multiple checkpoint molecules that inhibit T cell-mediated cytotoxicity, it also reduces expression of many adhesion molecules including E-cadherin, which is a known inhibitory ligand of NK cell receptor (KLRG1) [102]. Other well-established NK cell-activating molecules, such as PVR, are also upregulated upon EMT induction [103,104]. It is, therefore, not surprising that EMT may be accompanied by increased sensitivity to NK cell-mediated cytotoxicity [105]. Although the anti-tumor potential of CD8^+^ T cells is well accepted, the prognostic significance of their intratumoral homing is highly variable across different breast tumor subtypes [106]. The impact of CD8^+^ T cells in the tumor microenvironment (TME) on tumor epithelial-mesenchymal plasticity, on the interplay with other immune cells, and on associated metastatic traits in breast cancer cells are incompletely understood. Furthermore, it will be critical to clarify how the inflammatory TME and epithelial-mesenchymal plasticity influence CD8+ T cell activity and survival.

## 6. Impact of Immunosuppressive TME on ICBT

The intertumoral heterogeneity of the immune microenvironment dictates ICBT responses. We profiled the immune compartment in a wide variety of syngeneic triple-negative breast cancer (TNBC) models and discovered two prototypes of the immune microenvironment. In the first prototype, tumors induce systemic accumulation of neutrophils. These neutrophils overexpress multiple immunosuppressive pathways and may represent granulocytic myeloid-derived suppressor cells (gMDSCs). Macrophages co-exist but only constitute a minority of the myeloid cells in the tumor. In the second prototype, there does not appear to be an increase of neutrophils; rather, there is exclusively a local enrichment of tumor macrophages, which often (but not always) polarize toward the immunosuppressive M2 status. We have denoted these two “immunosubtypes” of mammary tumors as NES and MES, respectively. Apparently, NES and MES rely on different types of myeloid cells to escape immunosurveillance (data not shown). Indeed, when initially responsive MES tumors recurred neutrophils or gMDSCs accumulated, suggesting a conversion from MES toward NES or a switch of suppressor cell types. Depletion of neutrophils reduced this acquired ICBT resistance. Thus, immunosuppression may be exerted by different cell types in different tumor contexts.

EMT clearly contributes to the development of different immune microenvironments. Intriguingly, analyses of eight murine models representative of both NES and MES subtypes revealed that EMT contributes to the development of the dichotomous myeloid microenvironment. EMT has been linked to the recruitment of macrophages to the tumor microenvironment via chemokines like CCL2 [39,40]. Previous studies have also demonstrated that EMT drives expression of checkpoint ligands in cancer cells [51]. Taken together, the connection between EMT and cancer cell- or macrophage-mediated immunosuppression has been well established, which makes it an appealing therapeutic target to enhance immunotherapies. Inhibition of EMT, by targeting the EMT signaling pathways using TGF-β1 inhibitors while reverse immunosuppression by cancer cells and macrophages, may coincidentally cause accumulation of neutrophils, which can act as gMDSCs and lead to an alternative immunosuppressive mechanism, independent of checkpoints. Indeed, previous studies using syngeneic lung cancer models already indicate the existence of the dichotomous myeloid cell compartment in this cancer type as well [107]. Preclinical studies demonstrate that gMDSCs promote tumor progression through suppressing anti-tumor immunity [108,109] and promoting tumor-initiating cells (TIC) through the Notch pathway [109]. Interestingly, a recent study suggested that the former activity may depend on endogenous estrogen receptor alpha (ER) signaling [110], raising the possibility that endocrine deprivation therapies could be used for gMDSC elimination [111]. Endocrine deprivation therapies are standard-of-care for ER+ breast cancer.

All these findings suggest that EMT drives a switch of immunosuppression from gMDSC-mediated, checkpoint-independent mechanisms to macrophage/cancer cell-mediated, checkpoint-dependent mechanisms. Consequently, gMDSC accumulation may represent an escape pathway upon EMT inhibition that allows tumors to maintain an immunosuppressive microenvironment. Moreover, estrogen signaling and the Notch pathway mediate the pro-tumor effects of gMDSCs and provide potential therapeutic targets to eliminate these cells, a strategy that may complement anti-EMT treatment and ICBT.

## 7. Role of Bioinformatics

To identify potential relationships between phenotypic traits of tumors, including EMT and immune cell populations, and cancer outcomes, bioinformatics has long played a critical role. Gene expression profiles reveal the underlying biology of the tumors and can be used to dissect the features that are correlated with responses. A key observation is that signaling events lead to a transcriptional response, even if they are driven upstream by post-translational modifications. Thus, the signature of a pathway can be used to identify the molecular processes associated with clinical events.

Gene expression signatures involving biological processes have been generated using machine learning techniques. In short, the development of signatures is typically framed as a classification problem, where the goal is to identify a set of genes that can distinguish two biological states, such as epithelial or mesenchymal cells. To accomplish this, a training set is generated comprised of gene expression profiles of the two states. Then, differential expression analysis methods, such as DESeq2 [112] or EdgeR [113], are used to identify genes that can differentiate the two states. Finally, machine learning algorithms, such as SIGNATURE [114], can be used to score the expression profiles of new samples to provide a quantitative measure of the similarity to one or the other state.

Based on such approaches, an EMT signature has been linked to several outcomes including responses to chemotherapy [115], targeted therapies [116], patient survival [117,118], recurrence [119], and metastasis [120]. In addition to these outcomes, our studies and others have linked an EMT signature with markers or other evidence of immune modulation such as immune checkpoints [51] or low T cell infiltration in NSCLC [121]. Indeed, an increasing amount of evidence points to an association between EMT and the immune system in human tumors.

Independent of EMT, several bioinformatic analyses have supported the role of immune cells and clinical outcomes [122,123,124,125,126,127,128,129,130]. This demonstrates the ability of this technology to identify signatures of immune cells in bulk tumor samples. These analyses are enabled by the fact that the gene expression profiles of tumors reflect both the cancer cell and stromal/immune compartments. Although the signature of the immune cells can be seen in the bulk gene expression profiles, methods have been developed that can deconvolute the profiles into the constituent parts, enabling a more accurate quantification of the immune cell types that comprise the tumor [131,132].

One of the limitations of the prior studies is that they provide a limited resolution in quantifying the immune cell types (i.e., how many CD4+ T cells there are), and also in identifying the cell subtypes (i.e., are these Th1, Th2, or other CD4+ T cells). To address these questions, single-cell sequencing technologies that have the have the capacity to profile a range of cell types are rapidly being adopted. While powerful, these technologies have introduced a new bioinformatics challenge. The result of a single-cell RNA-Seq (scRNA-Seq) assay is the generation of transcriptional profiles of a likely heterogeneous immune cell population. Therefore, one step in the processing is to identify the cell types present in the population. While markers for immune cells have long been established for flow cytometry experiments, they are of more limited use in scRNA-Seq profiles due to issues such as drop-out, a phenomenon where a gene is not profiled due to factors including a lack of sensitivity in the assay; or ambiguities in the accepted markers. In the future, to address this, machine learning methods can be applied to identify immune cells from scRNA-Seq profiles. Nevertheless, a recent study has revealed a previously unknown range of T cell activation states within breast tumors [133]. Future studies using single-cell technologies, coupled with increasingly sophisticated bioinformatic analyses, will likely reveal new nuances in the relationships between the immune system, EMT, and cancer. Additional multiplex technologies will allow the spatial localization of these cells.

## 8. Conclusions

In this review, we have highlighted mechanistic vulnerabilities in mesenchymal tumor cells due to their ability to reprogram the tumor immune microenvironment. Because immunotherapy is rapidly emerging as a game-changing approach in many cancer types, including lung cancer, it will be critical to develop a greater understanding of those patients most likely to benefit and the mechanisms defining primary and acquired resistance. It will also be important to co-target EMT-related vulnerabilities along with the PD-L1/PD-1 immune checkpoint axis, given the large contribution of EMT to resistance mechanisms. Most importantly, the EMT phenotype has a distinct relationship with the immune microenvironment that can potentially be leveraged for a transformative clinical benefit for common epithelial tumors. EMT broadly up-regulates multiple immune checkpoint and inflammatory molecules to produce CD8+ T cell exhaustion, highlighting multiple potential mechanisms for the development of therapeutic resistance to immune therapy in mesenchymal tumors. EMT is a multidimensional process with different axes and these multiple parameters are exploited in malignancy under selective pressures including cytotoxic and biological therapies, hypoxia, energetics, and immune surveillance. As such, there is a pressing clinical need for targeted, biomarker-directed therapies to address both primary and acquired immunotherapy resistance. Incorporation of targeted agents (and validated biomarkers) that modulate immune suppression could expand the patient population that responds to immune checkpoint inhibitors and help address immunotherapy resistance. Studying these questions and integrating information across cancer types, instead of a single cancer, will shed light on combinatorial strategies that may be more generally applicable. Moreover, these comparative studies may also yield new insights since the treatment of cancer as evidenced in recent basket trials now is geared to understanding common vulnerabilities, e.g., mismatch repair deficiencies, as criteria for using ICBT across tumor types.
